# Aptamer‐conjugated mesoporous polydopamine for docetaxel targeted delivery and synergistic photothermal therapy of prostate cancer

**DOI:** 10.1111/cpr.13130

**Published:** 2021-10-02

**Authors:** Liang Dai, Dapeng Wei, Jidong Zhang, Tianyu Shen, Yuming Zhao, Junqiang Liang, Wangteng Ma, Limin Zhang, Qingli Liu, Yue Zheng

**Affiliations:** ^1^ Department of Urology The First Hospital of Qinhuangdao Qinhuangdao China; ^2^ State Key Laboratory of Medicinal Chemical Biology School of Medicine Nankai University Tianjin China; ^3^ Department of Gastroenterology The First Hospital of Qinhuangdao Qinhuangdao China

**Keywords:** aptamer, chemo‐photothermal therapy, docetaxel, mesoporous polydopamine, prostate cancer

## Abstract

**Objectives:**

It is imperative to develop efficient strategies on the treatment of prostate cancer. Here, we constructed multifunctional nanoparticles, namely AS1411@MPDA‐DTX (AMD) for targeted and synergistic chemotherapy/photothermal therapy of prostate cancer.

**Materials and Methods:**

Mesoporous polydopamine (MPDA) nanoparticles were prepared by a one‐pot synthesis method, DTX was loaded through incubation, and AS1411 aptamer was modified onto MPDA by the covalent reaction. The prepared nanoparticles were characterized by ultra‐micro spectrophotometer, Fourier transform infrared spectra, transmission electron microscope, and so on. The targeting ability was detected by selective uptake and cell killing. The mechanism of AMD‐mediated synergistic therapy was detected by Western blot and immunofluorescence.

**Results:**

The prepared nanoparticles can be easily synthesized and possessed excellent water solubility, stability, and controlled drug release ability, preferentially in acidic context. Based on *in vitro* and *in vivo* results, the nanoparticles can efficiently target prostate cancer cells, promote DTX internalization, and enhance the antitumor effects of chemo‐photothermal therapy strategies under the NIR laser irradiation.

**Conclusions:**

As a multifunctional nanoplatform, AS1411@MPDA‐DTX could efficiently target prostate cancer cells, promote DTX internalization, and synergistically enhance the antiprostate cancer efficiency by combining with NIR irradiation.

## Graphical abstract

Schematic diagram of AS1411@MPDA‐DTX‐mediated synergistic therapy for prostate cancer and the underlying mechanism.

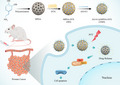

## 1 INTRODUCTION

Prostate cancer is one of the most common cancer among men and is the most frequently diagnosed cancer in more developed countries.^1^ According to the clinical scenario of the patient, surgical or nonsurgical treatments are available as curative therapy concepts, such as surgery, chemotherapy, radiation therapy, androgen deprivation therapy, and active surveillance. Although some improvements have been made in the treatments, nearly all diagnostic and therapeutic approaches for prostate cancer still rely on highly invasive and nonspecific techniques. Radical prostatectomy or radiotherapy are two main curative forms of therapy and are beneficial for the mortality of prostate cancer, however, surgery and radiation may result in serious outcome of incontinence and erectile dysfunction.^2^, ^3^ Chemotherapy is naturally palliative in the early stage, and among those chemotherapy agents, docetaxel (DTX) is the first to improve the overall survival in metastatic castration‐resistant prostate cancer.^4^, ^5^ Nevertheless, chemotherapy may cause many toxic side effects due to the high doses, frequent administration, and nonspecific targeting. Therefore, it is imperative to develop efficient, targeted, and noninvasive therapeutic strategies.

Recently, with the development of nanotechnology, nanoparticle‐based cancer diagnosis and therapy has attracted much attention.^6^, ^7^, ^8^, ^9^, ^10^, ^11^ Many nanoparticles have been employed to improve the delivery efficiency and sustained release of DTX, such as gold nanorods,^12^ poly(lactide‐co‐glycolide) (PLGA),^13^ mesoporous CuS,^14^ and mesoporous silica nanoparticles.^15^, ^16^ Mesoporous polydopamine (MPDA), spontaneously polymerized by dopamine, displays striking properties in optics, electricity, magnetics, biocompatibility, and biodegradability and has gained much attention for biomedical applications.^17^, ^18^ MPDA has been reported to load chemotherapy agents for enhanced anticancer therapy and reduced side effects.^19^, ^20^ In addition, MPDA shows strong near‐infrared (NIR) absorption, efficiently converts NIR light into heat to kill cancer cells, and finally realizes synergistic chemotherapy and photothermal therapy of cancers.^21^ Furthermore, benefit from the rich chemical groups, MPDA can be functionalized by many functional molecules, such as the targeted hyaluronic acid, amphipathic polyethylene glycol, and nucleophilic thiols.^22^, ^23^, ^24^

Surface nucleolin (NCL) is one of overexpressed protein on the surface of prostate cancer cells, and the inhibition of NCL can exert antineoplastic effects against prostate cancer cells.^25^, ^26^ AS1411, an aptamer, can specifically bind to NCL and displays cytotoxicity and antitumor activities. AS1411 has been widely used in targeted anticancer therapy.^27^ AS1411‐conjugated nanoparticles, such as carbon dots, gold nanoparticles, and chitosan nanoparticles, can selectively accumulated in cancer cells, achieving the enhanced the effect of loaded drugs and reduced side effects.^28^, ^29^, ^30^ However, aptamer AS1411‐modified MPDA to target prostate cancer has never reported. Herein, we designed a multifunctional nanoparticles based on MPDA for targeted delivery of DTX and synergistic chemo‐photothermal therapy against prostate cancer. DTX is encapsulated in the mesopores of MPDA with high payload and then modified the particles with AS1411 to target prostate cancer cells. The prepared nanoparticles, AS1411@MPDA‐DTX (AMD), can selectively enter prostate cancer cells and release DTX from the nanoparticles relies on the low pH of tumor microenvironment. The nanoparticles we designed possess drug delivery capabilities, cancer‐targeted properties, and photothermal conversion characteristics and provide potential candidates for enhanced antiprostate cancer therapy.

## 2 MATERIALS AND METHODS

### 2.1 Synthesis of AS1411@MPDA‐DTX nanoparticles

MPDA nanoparticles were prepared according to the previous reports.^31^ Briefly, 720 mg F127 and 834 µL TMB solution were added into the mixture of ethanol (120 ml) and H_2_O (130 ml) and stirring for 0.5 h. After that, 10 ml Tris solution (9 mg/ml) was added into the mixture; meanwhile, 60 mg dopamine was added and then stirred for 24 h at room temperature. Finally, MPDA nanoparticles were collected by centrifugation, and the template removal was performed by extraction method in ethanol/acetone mixture.

MPDA‐DTX nanoparticles were prepared by incubation. 100 mg MPDA nanoparticles and different concentrations DTX (50, 100, or 200 mg) were added into methanol and stirring for 24 h at room temperature. MPDA‐DTX nanoparticles were washed by double distilled (ddH_2_O) for twice and collected by centrifugation.

AS1411@MPDA‐DTX nanoparticles were synthesized by the covalent reaction between MPDA and carboxyl modified AS1411 aptamer. MPDA‐DTX nanoparticles were dispersed in ddH_2_O, and AS1411 was dissolved in DNase and RNase‐free water. 5 µL AS1411 solution was added into EDC•NHS solution for 30 min at 4℃, and then, MPDA‐DTX was added into the above mixture and stirring for 2 h. The final products were purified by centrifugation.

### 2.2 Characterization of AS1411@MPDA‐DTX nanoparticles

The UV absorbance of MPDA, DTX, AS1411, MPDA‐DTX, and AS1411@MPDA‐DTX were detected by UV‐Visible spectrophotometer (UV‐6000T). Fourier transform infrared (FT‐IR) spectra were conducted on FT‐IR spectroscopy (Nicolet‐5700). Dynamic light scattering (DLS) was employed to measure the size and zeta potential by Zetasizer Nano ZS90 analyzer (Malvern). Transmission electron microscope (TEM) images were taken by JEM‐2100F (JEOL) operated at an acceleration of 100 kV.

### 2.3 Release of DTX

10 mg of AS1411@MPDA‐DTX nanoparticles was dispersed in 2 ml Tween‐80 (0.1% w/v) and then transferred into a dialysis bag, which was dialyzed against 20 ml PBS with pH values of 5.2 and 7.4. The system was shaken at 37°C with a speed of 100 rpm under dark. At predetermined periods, 100 µl solution was withdrawn from the solution, and DTX quantities were detected by ultra‐micro spectrophotometer.

### 2.4 Photothermal performance

Different concentrations of AMD nanoparticles solution and ddH_2_O were dropped into 2‐ml EP tubes. All samples were irradiated by 808 nm continuous‐wave NIR laser (1 W/cm^2^) for 10 mins, and the temperature of solutions was obtained by an infrared imaging camera every 10 s. The photothermal conversion efficiency (*η*) of AMD nanoparticles was calculated according to previous method.^21^

### 2.5 Cellular uptake

PC‐3 cells and RWPE‐1 cells were incubated with AS1411@MPDA‐DTX nanoparticles for 24 h. All cells were collected through centrifugation and then washed twice with PBS. All samples were analyzed using a Attune NxT Flow Cytometer (Thermo Fisher). PC‐3 cells and RWPE‐1 cells after incubation with AS1411@MPDA‐DTX nanoparticles for 24 h were fixed by paraformaldehyde and then stained with phalloidin and DAPI. Fluorescence images were obtained using a confocal laser scanning microscopy (Nikon).

### 2.6 In vitro cytotoxicity

In vitro cytotoxicity of MPDA, DTX, MPDA‐DTX, and AS1411@MPDA‐DTX with or without irradiation was determined using the CCK‐8 assay and cell apoptosis assay. Briefly, PC‐3 cells incubated with different particles and then irradiated using 808 nm laser with an intensity of 100 mW·cm^−2^ for 5 min. Cell viability was determined by CCK‐8 kit, and OD value was measured at 450 nm. Cell apoptosis assay was detected using Annexin V–FITC apoptosis detection kit and analyzed with Attune NxT Flow Cytometer.

### 2.7 Cell death mechanisms

Generation of ROS was detected by a ROS probe, DCFH‐DA. Cells after different treatments were rinsed and then stained with DCFH‐DA and Hoechst33342, which was diluted in serum‐free medium. The fluorescence images were immediately obtained using a fluorescent inverted microscope (Leica).

Cell apoptosis‐related proteins were detected by Western blot and immunofluorescence. For Western blot, cells after different treatments were lysed in lysis buffer to harvest total proteins. Then, equal amounts of proteins were separated on SDS–polyacrylamide gels and transferred to a polyvinylidene difluoride membrane. The expression of Bax, Bcl‐2, and caspase‐3 was probed with anti‐Bax, anti‐Bcl‐2, and anti‐caspase‐3 antibody and secondary antibodies. GAPDH was used as a control. The bands were observed with a chemiluminescence imaging system after ECL color developing, and the quantitative analysis was calculated as the protein/GAPDH ratio.

For immunofluorescence, cells after different treatments were fixed with paraformaldehyde and then incubated with anti‐Bax, anti‐Bcl‐2, anti‐caspase‐3 antibody, and fluorescent secondary antibody. Before observed under confocal laser scanning microscopy, cells were stained with phalloidin and DAPI.

### 2.8 *In vivo* therapy

PC‐3 cells were seeded in BALB/c mice to develop prostate cancer model. In detail, PC‐3 cells were collected in a serum‐free medium, and 10^6^ cells were injected subcutaneously into the left flank of BALB/c mice. Mice were divided into 5 groups when tumors grew to 100 mm^3^. The control group was injected with normal saline, and the DTX group, MPDA‐DTX group, and AS1411@MPDA‐DTX group were injected intravenously with DTX, MPDA‐DTX, or AS1411@MPDA‐DTX separately every 48 h at equivalent DTX doses of 0.5 mg/kg. AS1411@MPDA‐DTX plus laser group was injected with AS1411@MPDA‐DTX every 48 h, and 4‐hr post‐injection, tumors were exposed to 808 nm NIR laser for 15 min with an intensity of 2 W·cm^−2^. The tumors size and mice weights were measured every 48 h, and at 14th day, all of the mice were euthanized. Tumors and major organs (heart, lung, liver, kidney, and spleen) were dissected and subjected to hematoxylin and eosin (H&E) staining.

### 2.9 Statistical analysis

The results were shown as mean ± SD. The significance of differences between groups was determined via one‐way analysis of variance (ANOVA), and significance of differences between two groups was determined via t test. All statistics were conducted using SPSS, and differences were considered significant if *p* < 0.05.

## 3 RESULTS

### 3.1 Synthesis and characterization of AMD

**FIGURE 1:**
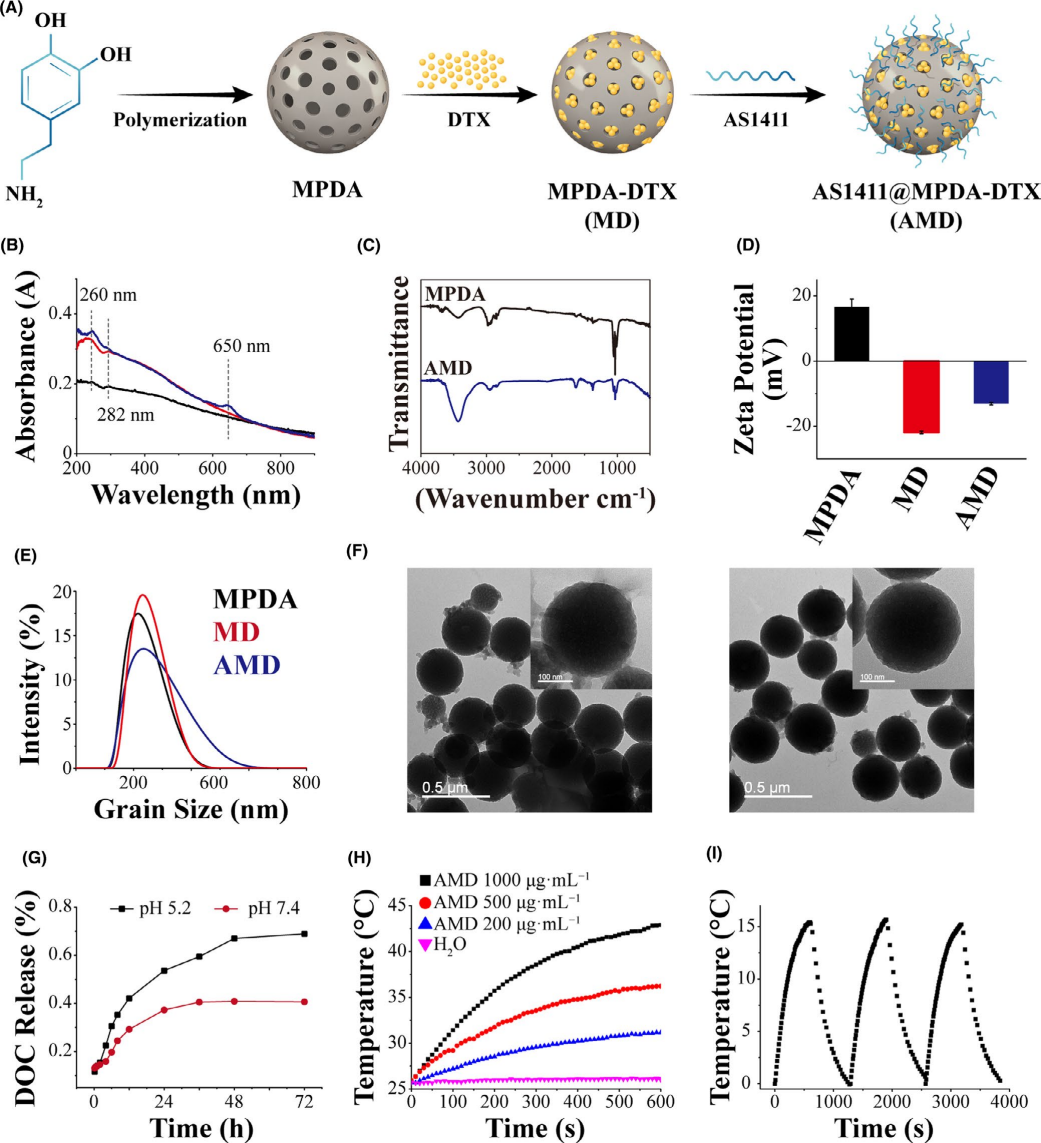
Synthesis and characterization of AMD. (A) Schematic illustration of synthesis of AMD. (B) Spectra of MPDA, MD, and AMD between 200 and 900 nm. (C) FTIR spectra of MPDA and AMD. (D) zeta potential of MPDA, MD, and AMD. (E) size distribution of MPDA, MD, and AMD. (F) TEM images of MPDA and AMD, scale bar: 0.5 μm and 100 nm. (G) In vitro DTX release profiles from MPDA. (H) Temperature elevation of AMD solutions at various concentrations during laser irradiation. (I) temperature elevation of AMD solutions at various concentrations during laser irradiation

The synthesis of AMD is shown in Figure 1A. Firstly, MPDA particles were synthesized by a facile one‐pot synthesis method referring to previously reported.^31^ DTX was loaded by incubation in methyl alcohol, and AS1411 was modified on MPDA through EDC/NHS coupling chemistry. The prepared nanoparticles showed a characteristic absorption peak of DTX (~282 nm) and AS1411 (~260 and 650 nm) (Figures 1B and S1 and S2), indicating the successful synthesis of AMD. FTIR spectra of MPDA and AMD show that the new peak generated at 1650 cm^−1^, which was due to the generation of the Schiff base (–C=N–) by the reaction of AS1411 and MPDA (Figure 1C). These results suggested that AS1411 was successfully conjugated on MPDA. The change of zeta potential also indicates the synthesis of AMD. MPDA possessed a positive charge, while DTX, AS1411, MD, and AMD were negatively charged (Figures 1D and S3). Zeta potential of AMD was about −13.07±0.37 mV, suggesting that AMD was relatively stable in water. The size of MPDA, MD, and AMD was detected by DLS, and AMD showed a little larger than MPDA or MD (Figure 1E). TEM images show that MPDA and AMD were uniform with good dispersion and spherical shape, and both MPDA and AMD displayed a mesoporous structure (Figure 1F). Besides, the diameters of these nanoparticles were insistence with DLS results.

The loading capacity of DTX in MPDA was detected. According to the standard curve and the drug‐loading amounts of DTX in Figure S4, the loading capacity of DTX in MPDA could reach about 1.85 mg/mg. Furthermore, the *in vitro* release of DTX from AMD under different pH conditions is shown in Figure 1G. AMD displayed a pH‐dependent DTX release in the neutral and acidic buffer, and DTX released more quickly in acidic buffer than neutral buffer. The light‐to‐heat conversion efficiency of AMD has been evaluated. As shown in Figure 1H, the temperature of 1000 μg/ml AMD solution was increased from 25°C to 42°C under laser irradiation in 600 s, and ddH_2_O did not show significant change under NIR laser irradiation. Furthermore, the photothermal heating effect of AMD nanoparticles exhibited concentration‐dependent (with particle concentrations from 200 to 1000 μg/ml). The photothermal conversion efficiency (η) of AMD nanoparticles was calculated to be 11% (Figure S5). In addition, after three cycles laser irradiation, the temperature changes of AMD solution under irradiation did not show obvious difference (Figure 1I), indicating the great photostability of AMD nanoparticles. Therefore, AMD nanoparticles possess a high photothermal effect and excellent photostability, which are favorable for PTT.

### 3.2 Selective uptake of AMD by PC‐3

**FIGURE 2:**
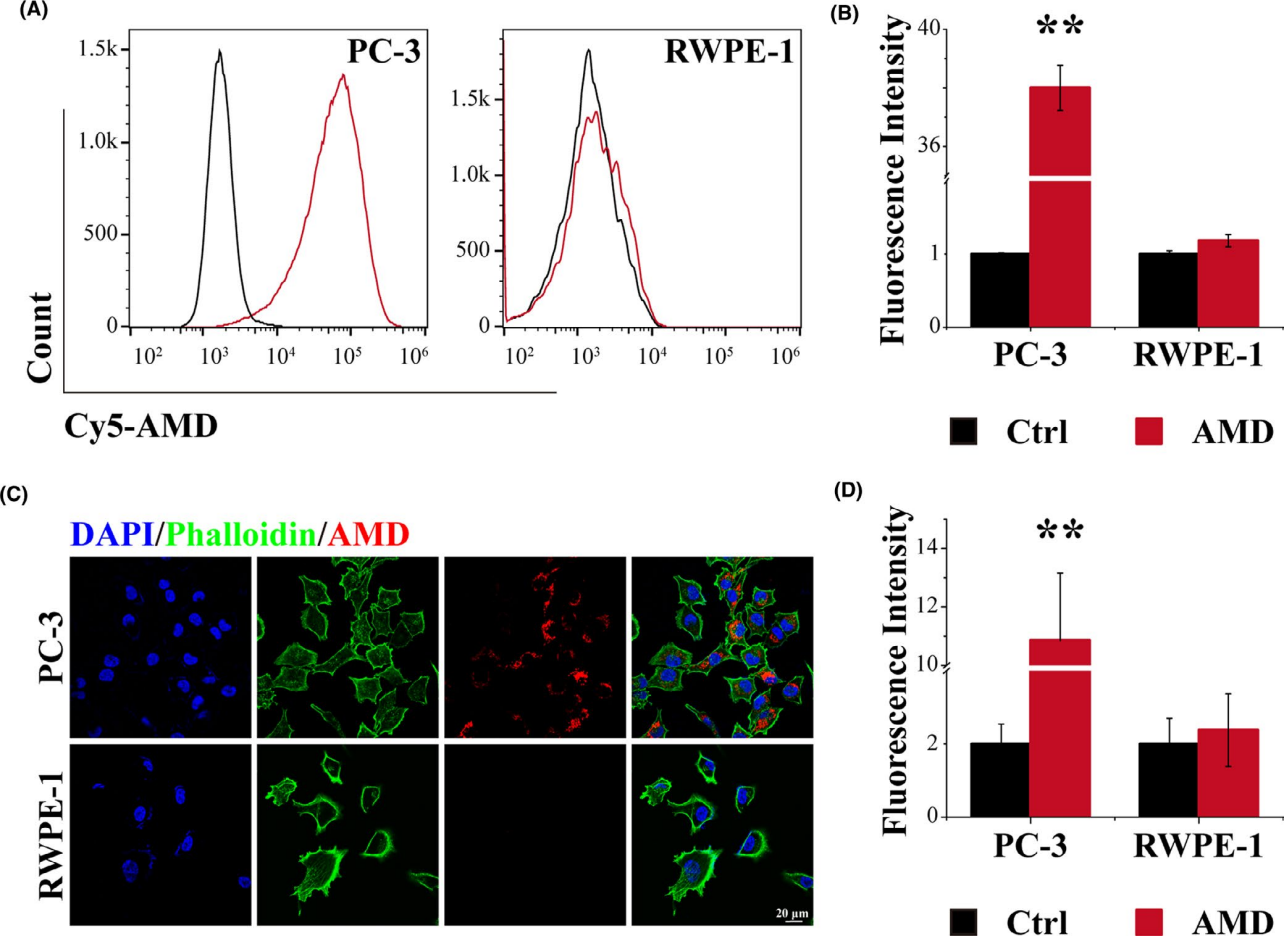
Selective uptake of AMD by PC‐3. (A) Flow cytometry results of PC‐3 and RWPE‐1. (B) quantitative analysis of flow cytometry results. (C) CLSM images of PC‐3 and RWPE‐1, scale bar: 20 μm. (D) quantitative analysis of CLSM images. Data are performed using one‐way analysis of variance (ANOVA) and presented as mean ± SD (*n* ≥ 3). Statistical analysis: * compared with control, **p < 0.01

The internalization of Cy5‐labeled AMD (Cy5‐AMD) in PC‐3 and RWPE‐1 was detected by flow cytometry and confocal laser scanning microscope (CLSM). As shown in Figure 2A, nearly all PC‐3 cells could uptake AMD particles, while RWPE‐1, a normal epithelial cell with low nucleolin expression level, could not uptake AMD particles. The fluorescence intensity of AMD in PC‐3 was significantly higher than control cells, while no significant differences in RWPE‐1 cells (Figure 2B). The preferential uptake of AMD by PC‐3 cells was also evaluated by CLSM and the quantitative analysis of red fluorescence (Figure 2C,D). PC‐3 showed more uptake than RWPE‐1, and the relative fluorescence intensity showed significant difference in PC‐3 cells. These results revealed that AMD could specifically target cancer cells, which overexpress nucleolin and reduce the uptake by normal cells.

### 3.3 Synergistic therapy of AMD *in vitro*

**FIGURE 3:**
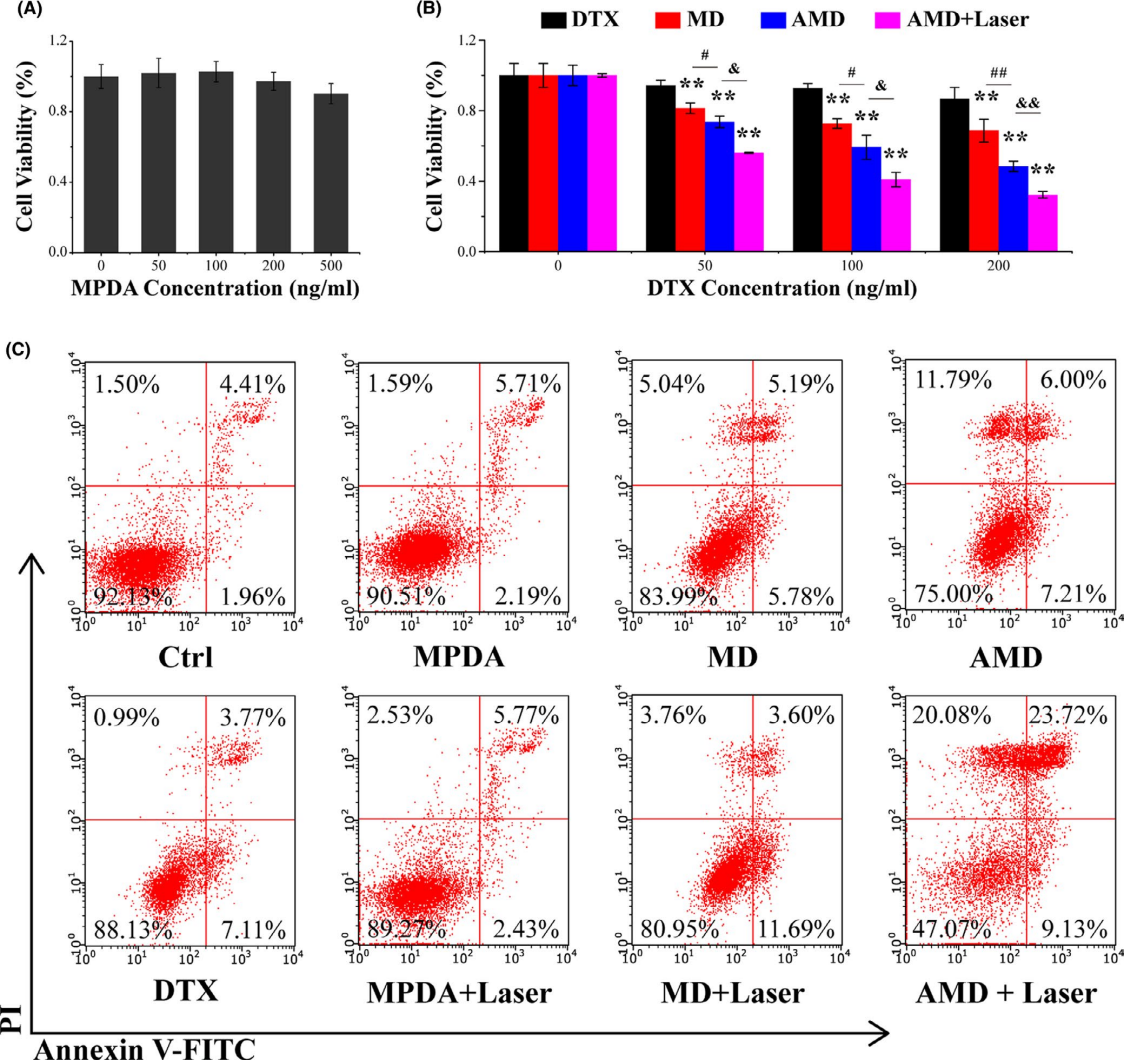
Synergistic therapy of AMD *in vitro*. (A) Cell viability of PC‐3 after different concentrations of MPDA incubation. (B) Cell viability of PC‐3 after different concentrations of DTX, MD, AMD incubation, and AMD incubation with irradiation. (C) Cell apoptosis of PC‐3 cells after different treatments. Data are performed using one‐way analysis of variance (ANOVA) and presented as mean ± SD (*n* ≥ 3). Statistical analysis: * compared with control, **p < 0.01; ^#^ *p* < 0.05, ^##^ *p* < 0.01. ^&^ *p* < 0.05, ^&&^ *p* < 0.01

To evaluate the biocompatibility and the antitumor effect of the synthesized nanoparticles, the vitality of PC‐3 cells after different treatments was detected through CCK‐8 assay. Given the importance of biosafety for nano‐drug delivery systems,^32^ the toxicity of blank MPDA was firstly examined. MPDA showed nearly negligible cytotoxicity against PC‐3 cells (Figure 3A), indicating the pretty biocompatibility of MPDA. Free DTX showed significant inhibition on the growth of PC‐3, and MPDA‐loaded DTX could obviously enhance the antitumor effect (Figure 3B). AS1411‐modified nanoparticles could significantly target PC‐3 cells and enhance the cancer killing efficiency than MPDA‐DTX (Figure 3B). In addition, irradiation could enhance the destruction of AMD‐incubated PC‐3 cells in vitro. These results indicated that AMD could promote the delivery of DTX and synergistic with PDT to enhance the antitumor effect. To further evaluate the therapeutic effect of the synthesized nanoparticles, the cell apoptosis of PC‐3 cells was analyzed by flow cytometry. Cells incubated with DTX showed increased apoptotic cells than control cells, and MD incubation could increase the apoptotic cells in some extent, about 16%, while AMD treatment induced about 25% cell death (Figure 3C). The differences in cytotoxicity between MD and AMD was ascribed to the targeting uptake of AMD to nucleolin‐positive cells. AMD plus irradiation could induce about 53% cell death, much higher than AMD alone, indicating that the synergistic treatment could significantly cancer cells. Furthermore, cells incubated with MPDA shows no significant difference with control cells, also indicating the good biosafety of MPDA.

### 3.4 Mechanism underlying the synergistic antitumor effect

**FIGURE 4:**
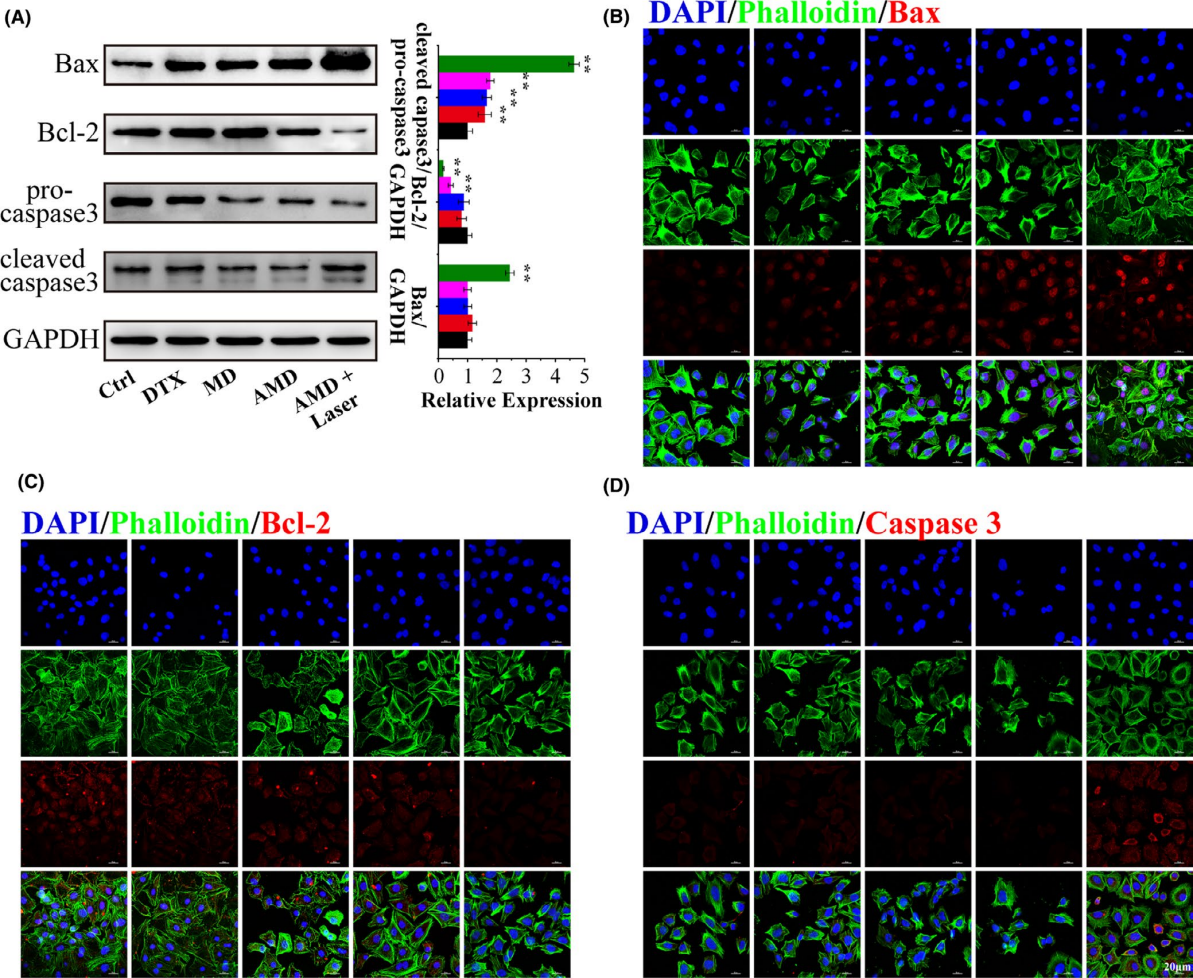
Mechanism underlying the synergistic antitumor effect. (A) Expression of Bax, Bcl‐2, and caspase‐3 detected by Western blot, and the quantitative analysis of Western blot results. (B) immunofluorescence images of Bax. (C) immunofluorescence images of Bcl‐2. (D) immunofluorescence images of caspase‐3. Scale bar: 20 μm. Data are performed using one‐way analysis of variance (ANOVA) and presented as mean ± SD (*n* ≥ 3). Statistical analysis: * compared with control, *p < 0.05, **p < 0.01; ^#^ *p* < 0.05, ^##^ *p* < 0.01

The ROS in PC‐3 after different treatments was examined by DCFH‐DA probe. Through fluorescent inverted microscopy observation, obvious production of ROS in PC‐3 cells was identified after AMD plus irradiation treatment, while the cells with AMD incubation show slight fluorescence (Figure S6). ROS is reported to induce the mitochondria‐mediated cell apoptosis; hence, the related proteins, Bcl‐2, Bax, and Caspase 3, were analyzed with Western blot and immunofluorescence. As displayed in Figure 4A, AMD‐incubated cells and AMD plus irradiation‐treated cells showed obviously decreased expression of Bcl‐2 and increased expression of Bax. The Western blot results and quantitative analysis show that AMD mediated synergistic therapy could significantly upregulate the expression of caspase‐3, indicating the activation of caspase cascades (Figure 4A). Immunofluorescence images also show that the pro‐apoptotic protein Bax and caspase‐3 was increased, while anti‐apoptotic protein Bcl‐2 was decreased after AMD treatment or AMD plus irradiation treatment (Figure 4B,D). These results illustrated that AMD‐mediated synergistic therapy could induce cell death through mitochondrial apoptotic pathway.

### 3.5 Synergistic therapy of AMD *in vivo*

**FIGURE 5:**
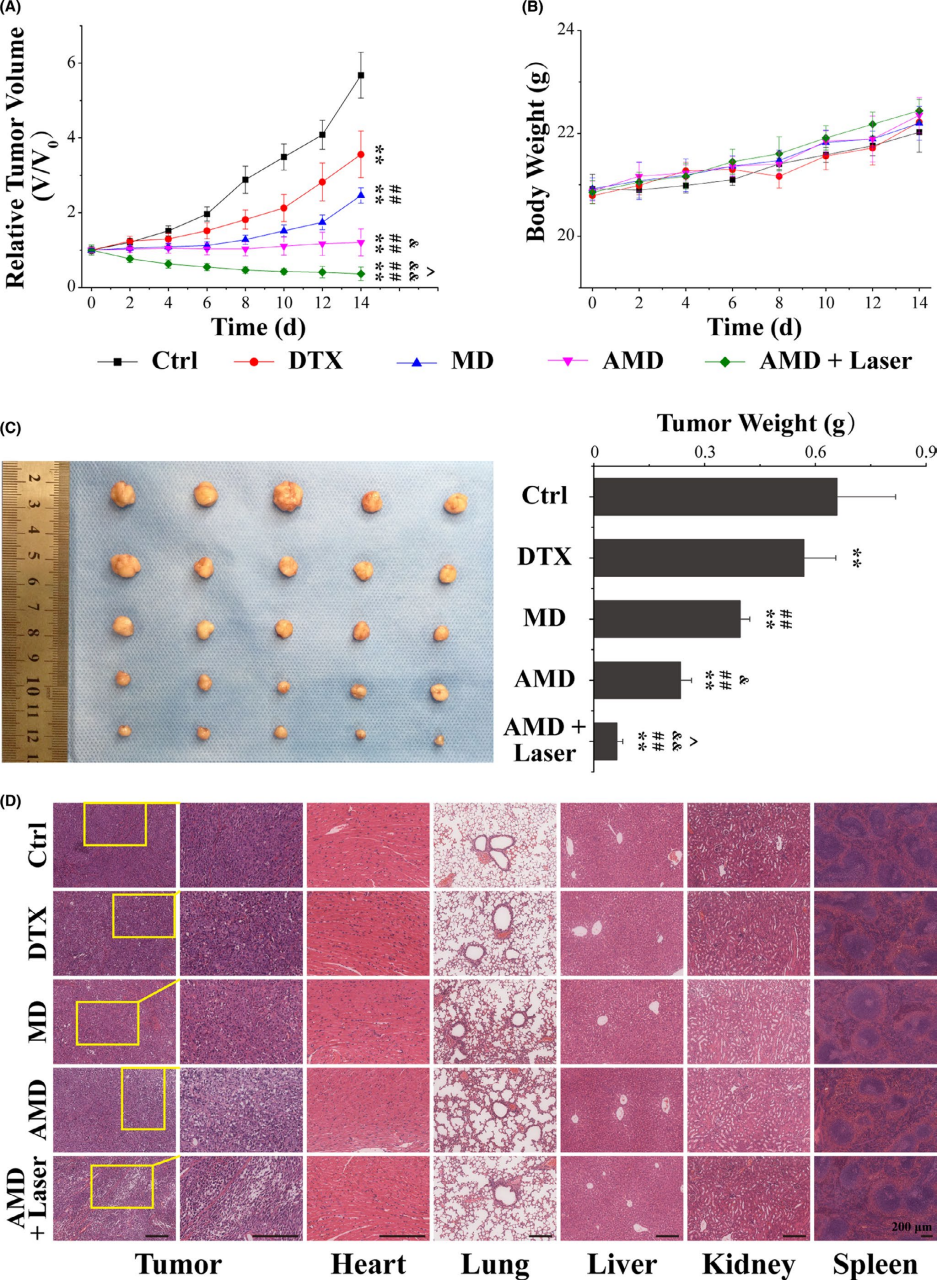
Synergistic therapy of AMD *in vivo*. (A) Relative tumor volume of PC‐3 tumor‐bearing mice with different treatments. (B) Body weight of tumor‐bearing mice with different treatments. (C) Photographs and weight of tumors dissected from each group on the 14th day after different treatments. (D) H&E staining images of tumor tissues (the second column are a magnified portion of images in the first column) and major organs (heart, liver, spleen, lung, and kidney) dissected from each group on the 14th day after different treatments. Scale bars: 100 μm. Data are performed using one‐way analysis of variance (ANOVA) and presented as mean ± SD (*n* ≥ 3). Statistical analysis: * compared with control, **p < 0.01; ^#^ compared with DTX group, ^#^ *p* < 0.05, ^##^ *p* < 0.01; ^&^ compared with MD group, ^&^ *p* < 0.05, ^&&^ *p* < 0.01; ^ compared with AMD group, ^*p* < 0.05, ^^*p* < 0.01

Inspired by the excellent *in vitro* anticancer efficient of AMD nanoparticles, the *in vivo* therapy efficacy was evaluated in PC‐3 tumor‐bearing mice model. The nanoparticles were injected by tail vein, and 808 nm laser was given at 4 h post‐injection. As displayed in Figure 5A, the control group shows a rapid tumor growth, and DTX‐ or MD‐treated mice shows a slower tumor growth than control group. Tumors in AMD group show nearly no significant increase in tumor volume, and AMD plus laser group shows an obvious inhibition of tumor growth. These results indicated that the therapeutic effect of single chemotherapy was limited, and the synergistic effect of chemotherapy and PTT was much more effective. In addition, the irradiation intensity was about 1 W/cm^2^, suggesting the extraordinary antitumor effect of AMD nanoparticles could be realized at a low power density. The digital photographs of tumors on the 14^th^ day after treatment also show that tumors in AMD group or AMD plus laser group were smaller than other groups, and tumors in AMD plus laser group were almost ablated (Figure 5C). The tumor weight in AMD plus laser group shows significant difference with other groups, which agree fairly well with the relative tumor volume. These results suggesting that AMD‐mediated synergistic therapy could efficiently inhibit the growth of prostate cancer. What is more, no apparent weight loss was seen in all groups (Figure 5B), illustrating that AMD nanoparticles did not induce systemic toxicity. Subsequently, H&E staining of tumor tissues was performed to further determine the antitumor effect of AMD nanoparticles. As shown in Figure 5D, cancer cells were lined up with hyperchromatic nuclei and mitotic figures in control group, while most cancer cells were destroyed in AMD group and AMD plus laser group. The results were consistent with the tumor volume and weight, indicating that AMD‐mediated synergistic therapy could induce most cancer cell death and tumor ablation. Moreover, the biocompatibility of AMD nanoparticles was also evaluated by histological analysis. The major organs, such as heart, lung, liver, spleen, and kidney, in AMD group and AMD plus laser group showed no obvious damage or inflammatory compared to control group, further verifying the biosafety of AMD nanoparticles (Figure 5D). These results suggested that AMD‐mediated synergistic therapy of chemotherapy and PTT could efficiently inhibit the growth of prostate cancer.

## 4 DISCUSSION

MPDA, one of polydopamine‐based nanoparticles, has been demonstrated to be appealing agents due to the porous structure, excellent biocompatibility, and photothermal stability.^33^, ^34^, ^35^ MPDA nanoparticles were widely applied in combination therapy. MPDA nanoparticles provided a versatile platform for drug delivery in favor of the large surface area and high pore volume. Previous studies showed that MPDA tends to have a high loading capacity^17^, ^36^; here, the loading capacity of DTX in MPDA could reach about 1.85 mg/mg. The high loading capacity might be on account of the mesoporous structure of MPDA and the strong electrostatic attraction between the negative charge of DTX and the positive charge of MPDA surface.^37^ The release of DTX from MPDA nanoparticles was pH responsive. Considering the tumor environment are more acidic than the normal tissues,^38^ the pH‐sensitive release was ideal. The pH‐sensitive system can reduce the drug release in normal tissues to inhibit the toxic side effect, but release in tumor tissues and consequently enhance the antitumor efficiency.

To specially target cancer cells, nanoparticles were modified with various targeting ligands. Polydopamine‐based nanomaterials possessed a multifunctional surface since there are a large number of modifiable sites, ligands such as antibodies, peptides, nucleic acids, and small molecules could be modified onto the surface of these nanomaterials to improve the specificity of drug delivery and enhance therapeutic efficiency. Cheng et al modified FA onto PDA capsules to fabricate DOX‐loaded FA‐PEI‐PDA capsules to enhance the endocytosis of DOX by HeLa cells.^39^ Nucleolin is a multifunctional protein participated in many modulation in the nucleolus, cytoplasm, and cell membrane, and nucleolin can bind to various ligands and regulate physiological functions.^40^, ^41^, ^42^ Previous researches also suggested that the expression of nucleolin is not normal in prostate cancer, and the elevated expression of nucleolin is related to a series abnormity, such as carcinogenesis, proliferation, survival, and metastasis of cancer cells.^43^, ^44^ Hence, nucleolin may be a promising target for antiprostate cancer therapy. AS1411, a 26‐mer DNA aptamer, can specifically bind to nucleolin.^45^ The aptamer serves as not only nucleolin targeting ligand but also therapeutic agent, it has been proved to selectively enter target cells and inhibit the growth of cancer cell lines in vitro.^46^ Besides, many drug molecules can covalently bind to AS1411 via simple conjugation strategy, and AS1411 can be grafted onto the surface of different nanocarriers, such as polymeric micelles,^47^ DNA nanostructures,^48^ metal nanoparticles,^49^ and mesoporous silica nanoparticles^50^; thus, AS1411 has been widely used in targeted drug delivery and antiprostate cancer treatment.^51^, ^52^, ^53^ Here, AS1411 was employed to modify drug‐loaded MPDA to enhance cancer cell uptake and anticancer efficiency. The results showed that AS1411‐modified nanoparticles showed a selective uptake by PC‐3, while normal cell RWPE‐1 represented reduced uptake of AMD. Furthermore, AMD showed stronger tumor‐killing effect than MD nanoparticles. These results revealed that AMD could specifically target prostate cancer cells and efficiently induce the ablation of cancer cells.

Since AMD plus irradiation induced cell death mainly through cell apoptosis, the cell apoptosis pathway was detected. Photosensitization usually causes the generation of reactive oxygen species (ROS),^54^ and AMD plus irradiation treatment induced obvious generation of ROS, which was reported to induce the mitochondria mediated cell apoptosis. Bcl‐2 and Bax belong to Bcl‐2 family proteins, and Bcl‐2 is one of anti‐apoptotic proteins and Bax is one of pro‐apoptotic proteins.^55^, ^56^ The imbalance between Bcl‐2 and Bax plays an important role in mitochondria‐mediated cell apoptosis. AMD‐mediated synergistic treatment could notably induce the upregulation of Bcl‐2 and downregulation of Bax, causing the imbalance between the two proteins, increasing mitochondrial outer membrane permeabilization, releasing pro‐apoptotic factors, and finally activating caspase cascades.^57^, ^58^, ^59^ It was revealed that caspase‐3 was an “initiator” caspase, and the cleavage of caspase‐3 could result in dysfunction of many proteins and eventually the cellular changes associated with apoptosis.^60^ Our results showed that AMD‐mediated synergistic treatment could significantly induce the activation of caspase‐3, indicating that AMD‐mediated synergistic therapy could efficiently induce cell death through mitochondrial apoptotic pathway.

## 5 CONCLUSION

In conclusion, we have constructed an aptamer‐modified MPDA delivery system for targeted delivery and synergistic photothermal therapy of prostate cancer. The prepared nanoparticles, AS1411@MPDA‐DTX, could efficiently target prostate cancer cells and promote DTX internalization. The targeting ability may help the delivery system accumulate at tumor sites, and the controlled release ability of drugs, preferentially in acidic context, may enhance the antitumor efficient of DTX. In addition, NIR irradiation could mediate a photothermal therapy, which synergistically enhance the antiprostate cancer efficiency of the nanoparticles. Such a nano‐system may pave an avenue for the development of synergistic chemo‐photothermal therapy of prostate cancer. In the next, we will combine the previous research to optimize the nanoparticles, giving it excellent tumor‐affinity and the theranostic function to realize targeted diagnosis and therapy.

## CONFLICTS OF INTEREST

There are no conflicts to declare.

## AUTHOR CONTRIBUTIONS

LD, DW, and YZ designed the project. LD, DW, JZ, and TS performed the experiments and collected the data. YZ, JL, and WM analyzed the data, and LZ and QL organize the figures. LD drafted the manuscript and DW polished the manuscript. All authors read and approved the final manuscript.

## Supporting information

Figures S1‐S6

## Data Availability

The data used to support the findings of this research are from the public database, and the website has been provided in the corresponding position of the manuscript. The experiment data are available upon reasonable request.
