# Supplementary material for: Aptamer‐conjugated mesoporous polydopamine for docetaxel targeted delivery and synergistic photothermal therapy of prostate cancer
Source: Cell Prolif. 2021 Oct 2;54(11):e13130. doi: 10.1111/cpr.13130 (PMC8560597; doi:10.1111/cpr.13130)
Supplement: Supplementary file 1 — Figures S1‐S6 [file CPR-54-e13130-s001.docx]

Supporting Information

**Aptamer-Conjugated Mesoporous Polydopamine for Docetaxel Targeted Delivery and Synergistic Photothermal Therapy of Prostate Cancer**

Liang Dai^a‡^, Dapeng Wei^a‡^, Jidong Zhang^a^, Tianyu Shen^b^, Yuming Zhao^a^, Junqiang Liang^a^, Wangteng Ma^a^, Limin Zhang^a^, Qingli Liu^a^, Yue Zheng^c^*

^a^ Department of Urology, The First Hospital of Qinhuangdao, Qinhuangdao, 066000, China

^b^ School of Medicine, State Key Laboratory of Medicinal Chemical Biology, Nankai University, Tianjin, 300071, China

^c^ Department of Gastroenterology, The First Hospital of Qinhuangdao, Qinhuangdao, 066000, China

***CORRESPONDING AUTHOR:**

Yue Zheng

Department of Gastroenterology, The First Hospital of Qinhuangdao, Qinhuangdao, 066000, China

E-mail address: [zhengyue0335@126.com](mailto:%20zhengyue0335@126.com)

‡ Liang Dai and Dapeng Wei contributed equally to this study.


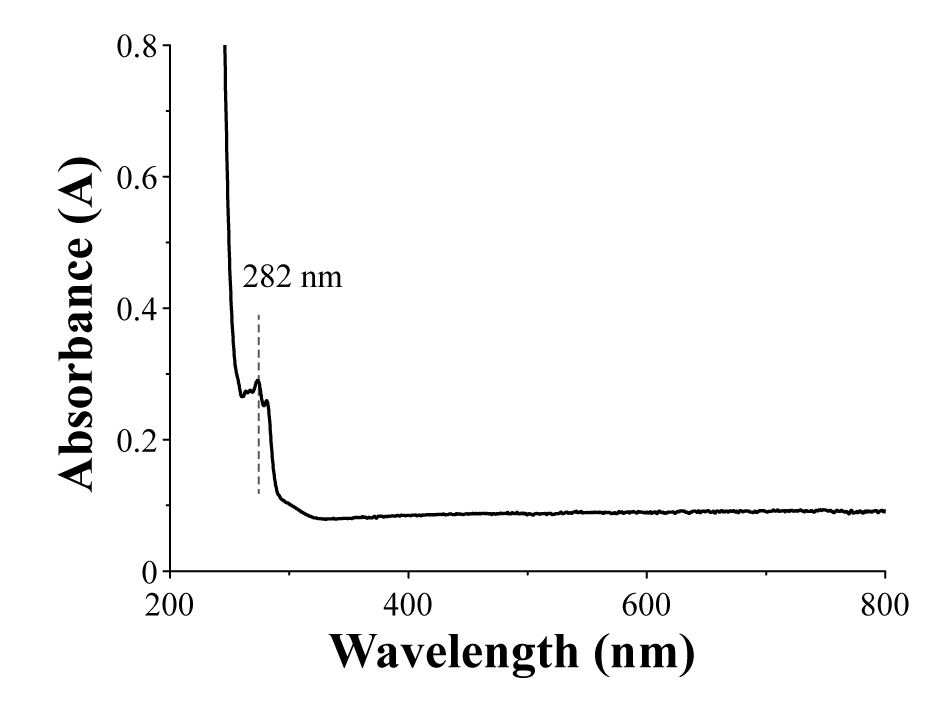


Fig. S1. Spectra of DTX between 200 and 800 nm.


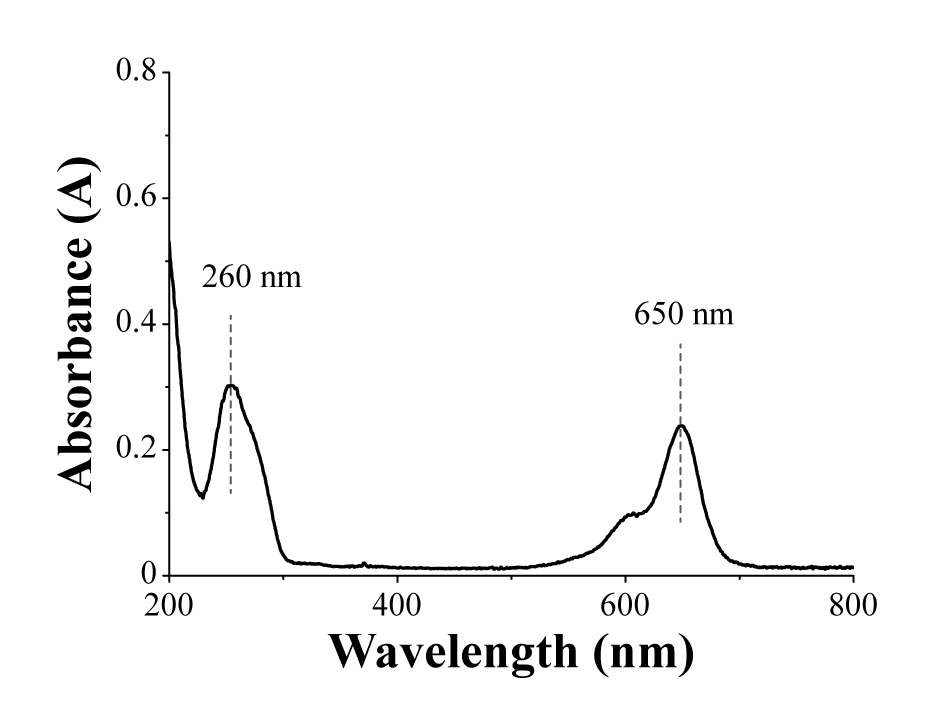


Fig. S2. Spectra of AS1411 between 200 and 800 nm.


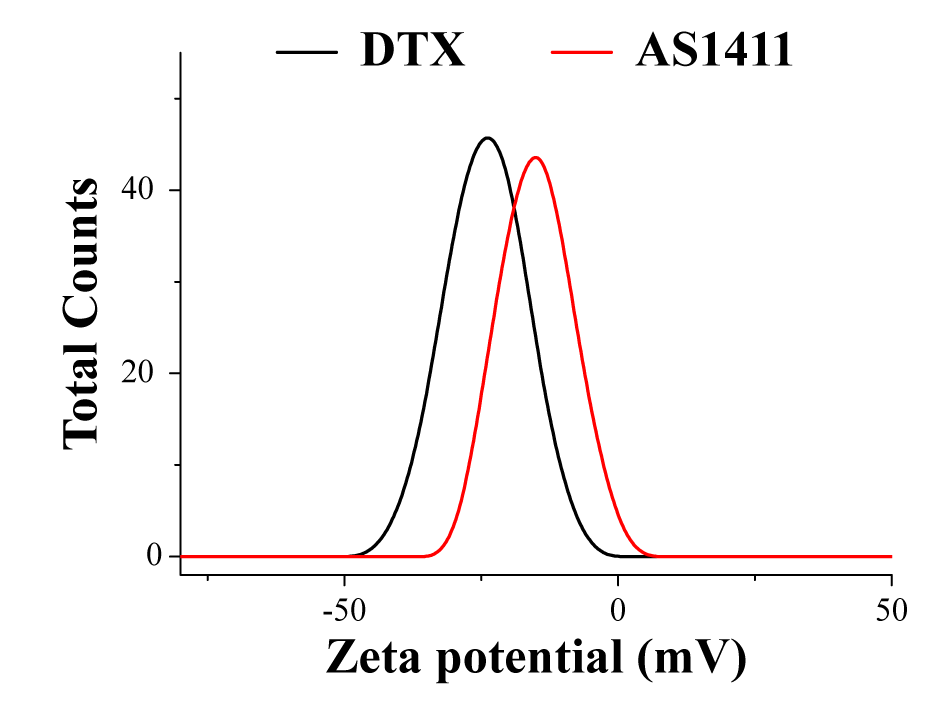


Fig. S3. Zeta potential of DTX and AS1411.


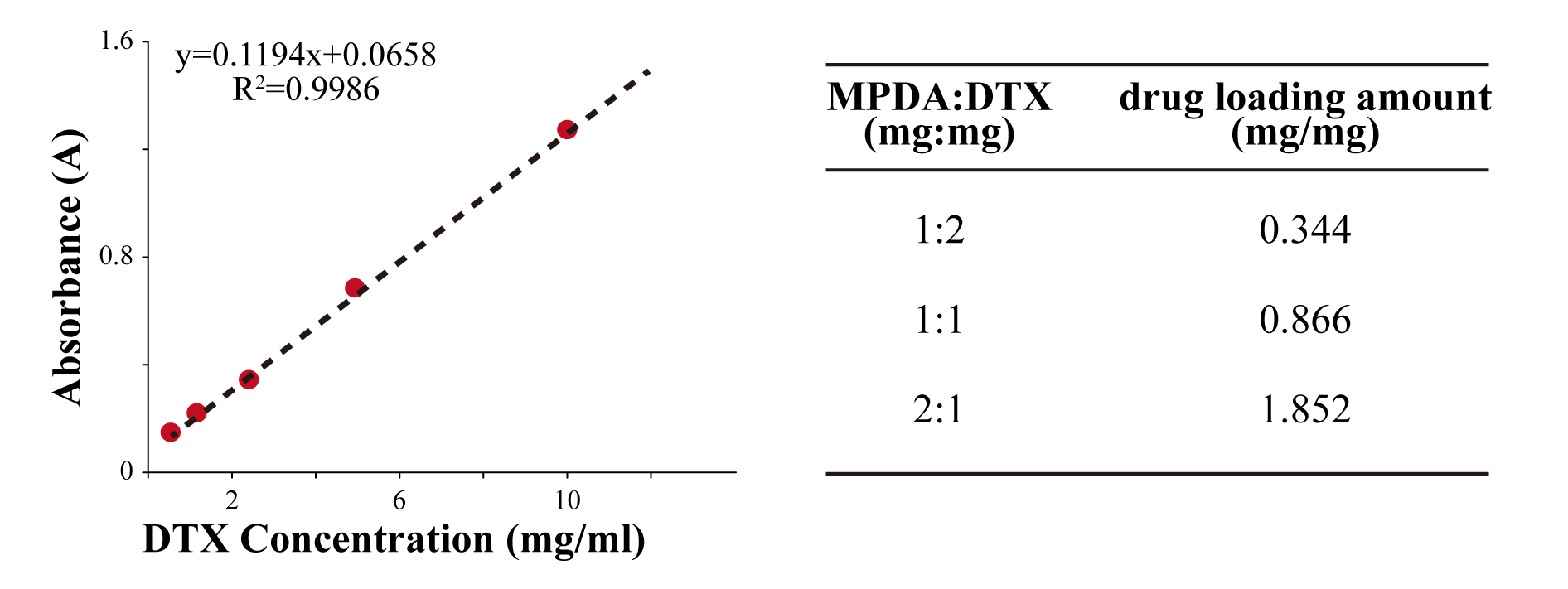


Fig. S4. Standard curve of DTX and the drug-loading amount of DTX in MPDA.


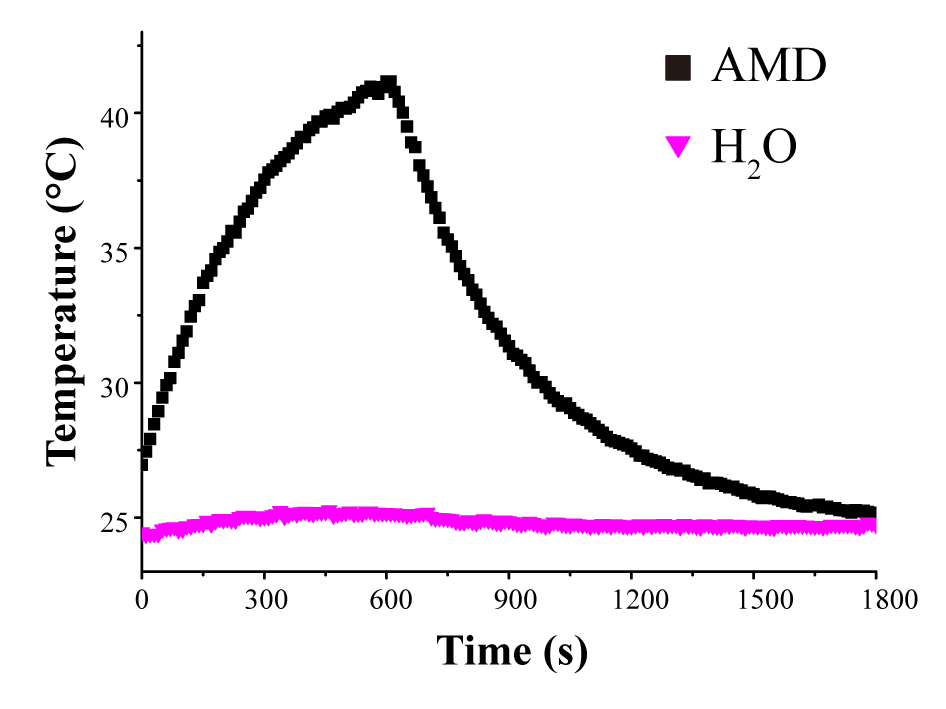


Fig. S5. Photothermal effect of AMD solution (1000 μg·mL^−1^) with laser irradiation (808 nm, 1 W·cm^−2^) for 10 min and left to cool down then.


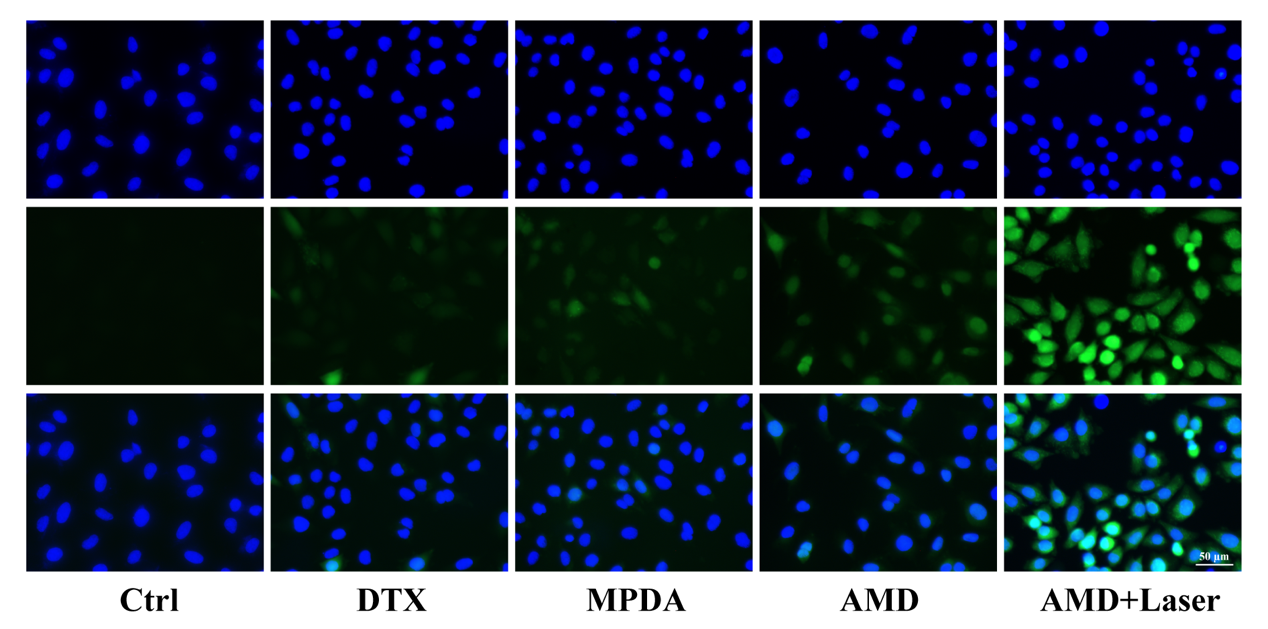


Fig. S6. Detection of ROS by DCFH-DA. Scale bar: 50 μm.
